# Supplementary material for: Micronutrient dynamics and deficiency risk across pregnancy and postpartum in a Slovak cohort
Source: PLoS One. 2025 Sep 4;20(9):e0331125. doi: 10.1371/journal.pone.0331125 (PMC12410874; doi:10.1371/journal.pone.0331125)
Supplement: Attachment — Submitted filename: Response to Reviewer3.docx [file pone.0331125.s001.docx]

Dear Dr. Abeywickrama,

thank you for the opportunity to revise our manuscript, *“Micronutrient Dynamics and Deficiency Risk Across Pregnancy and Postpartum in a Slovak Cohort”* [PONE-D-25-23187R1]. We are grateful for Reviewer 3’s constructive feedback and have addressed each point in detail below (in blue).

**Reviewer Comment 1:** *Clearly state whether supplement use, BMI, or parity were included as covariates or excluded from statistical analysis.*

**Response:** We appreciate the reviewer’s emphasis on clarifying covariate inclusion. While we collected self-reported data on supplement use during pregnancy, the variability in supplement composition (brand-dependent nutrient content) and inconsistent timing or duration of intake (e.g., folate use only during early pregnancy) precluded its meaningful inclusion as a covariate. BMI data were available for pregnant participants, but not for the non-pregnant control group, making comparisons inconsistent. Moreover, BMI during pregnancy presents interpretive challenges due to gestational weight changes. Parity data were not collected. As such, supplement use, BMI, and parity were excluded from statistical modeling.

This has now been explicitly clarified in the Methods section with the following sentence:

“Supplement use, BMI, and parity were excluded as covariates due to missing or inconsistent data across groups, high variability in supplement intake, and the challenge of standardizing BMI in the context of pregnancy.”

**Reviewer Comment 2:** *Conduct a light language edit to address grammatical inconsistencies and remove redundancy.*

**Response:** We have performed a thorough language review to improve clarity, eliminate grammatical inconsistencies, and remove redundant phrasing. Specific corrections include simplification of long sentences in the Discussion and removal of typographical repetitions such as “Ethics Committee Ethics Committee.”

**Reviewer Comment 3:** *Avoid causal terms like “decline” or “increase” since the study is cross-sectional. Instead, say “lower levels observed in group X” or “group differences.”*

**Response:** We fully agree with this point. The manuscript has been revised to remove causal terminology. Phrases such as “increase” or “decline” have been replaced with observational descriptors like “higher levels observed,” “group differences,” or “variation across groups” to reflect the cross-sectional design of the study accurately.

We believe these revisions address the reviewer’s concerns and have improved the clarity and scientific rigor of the manuscript. We appreciate the reviewer’s thoughtful assessment and hope the revised version meets the journal’s criteria for publication.

Sincerely,
Norbert Zilka,

On behalf of all co-authors
